# HIVed, a knowledgebase for differentially expressed human genes and proteins during HIV infection, replication and latency

**DOI:** 10.1038/srep45509

**Published:** 2017-03-30

**Authors:** Chen Li, Sri H. Ramarathinam, Jerico Revote, Georges Khoury, Jiangning Song, Anthony W. Purcell

**Affiliations:** 1 Infection and Immunity Program, Biomedicine Discovery Institute, Monash University, Melbourne, VIC 3800, Australia; 2 Department of Microbiology, Monash University, Melbourne, VIC 3800, Australia; 3 Department of Biochemistry and Molecular Biology, Monash University, VIC 3800, Australia; 4 Monash Bioinformatics Platform, Monash University, Melbourne, VIC 3800, Australia; 5 Department of Microbiology and Immunology, Peter Doherty Institute for Infection and Immunity, The University of Melbourne, Parkville, VIC 3010, Australia,; 6 Monash Centre for Data Science, Faculty of Information Technology, Monash University, Melbourne, VIC 3800, Australia

## Abstract

Measuring the altered gene expression level and identifying differentially expressed genes/proteins during HIV infection, replication and latency is fundamental for broadening our understanding of the mechanisms of HIV infection and T-cell dysfunction. Such studies are crucial for developing effective strategies for virus eradication from the body. Inspired by the availability and enrichment of gene expression data during HIV infection, replication and latency, in this study, we proposed a novel compendium termed HIVed (HIV expression database; http://hivlatency.erc.monash.edu/) that harbours comprehensive functional annotations of proteins, whose genes have been shown to be dysregulated during HIV infection, replication and latency using different experimental designs and measurements. We manually curated a variety of third-party databases for structural and functional annotations of the protein entries in HIVed. With the goal of benefiting HIV related research, we collected a number of biological annotations for all the entries in HIVed besides their expression profile, including basic protein information, Gene Ontology terms, secondary structure, HIV-1 interaction and pathway information. We hope this comprehensive protein-centric knowledgebase can bridge the gap between the understanding of differentially expressed genes and the functions of their protein products, facilitating the generation of novel hypotheses and treatment strategies to fight against the HIV pandemic.

Over 32 million are infected with HIV and with over 2 million new infections every year; it is still a major public health burden (UNAIDS 2010; http://www.unaids.org/globalreport/Global_report.htm). HIV predominantly infects CD4+ T-cells and leads to their death, impairing T helper immune responses during progressive infection and in the majority of cases leads to immunodeficiency if untreated^1^,^2^,^3^,^4^,^5^. While there is no effective vaccine or cure, administration of antiretroviral therapy reduces plasma viral loads in patients and greatly enhances their quality of life^6^. The therapy is life-long and cessation leads to return of significant viral loads predominantly from latent cellular reservoirs that include macrophages, dendritic cells, and particularly CD4+ T cells^7^,^8^,^9^. The implementation of effective strategies to clear HIV from body or to develop novel therapeutic interventions is contingent on understanding host cell changes during viral infection and latency. Gene arrays and transcriptomic analyses shed light on how HIV hijacks cell machinery and helped identify over 200 host factors that are crucial for HIV replication^10^,^11^,^12^,^13^,^14^,^15^. The advent of high-throughput next generation sequencing, proteomic and microarray analysis has generated large sets of data that were instrumental in revealing the role of HIV in modulation of expression of host genes (reviewed in Mehla *et al*.^14^).

In addition to development of drug interventions to control HIV infection, these studies can also point in the direction of methods to flush out the sources of latent virus. These remarkably stable reservoirs are established within a few days of HIV infection and can persist for years^16^ with an estimated half-life of around 43 months^17^. Elimination of these latent reservoirs is one of the greatest hurdles in the eradication of the virus^18^ and their analysis is crucial to understanding the mechanisms that support latency and to evaluate the effectiveness of agents that can reverse latency. Due to the rarity of latently infected cells in patients, several *ex vivo* and cellular models have been developed (reviewed in the study of Spina *et al*.^19^) to analyse the latent reservoirs. The detailed analysis of these models using high-throughput transcriptomic and genomic approaches has advanced our understanding of viral transcriptional silencing and led to the accumulation of a large and valuable pool of data from disparate sources^12^,^15^,^20^,^21^,^22^,^23^,^24^,^25^,^26^,^27^. Studying the mechanisms of action of existing LRAs (Latency-Reversing Agents) could shed light on newer therapeutic interventions by comparing host cell expression data from different cellular models and LRAs.

At present, most of these data are hosted on individual websites: Litchi^21^, Peachi^28^ or in collections^29^, which mainly focus on annotating the genome and expression of related genes during HIV infection, replication, and/or latency, with little information on the functional annotations of their protein products. In this study, we implemented a novel knowledgebase, termed HIVed, to provide a comprehensive curation of related human proteins, whose genes have been characterized to be differentially expressed during HIV infection, replication, and latency. This protein-centric database collected experimental data of differentially regulated genes from a variety of studies, creating the comprehensive database that covers a wide range of experimental studies and hence bridges the knowledge annotation gap between known differentially expressed human genes, their corresponding protein products, and functional annotations during the HIV infection, replication, and latency.

## Database construction and utility

### Data collection

In order to collect the mainstream experimental studies of gene expression during HIV infection, replication and latency, we searched the literature and extracted eleven published genomic and proteomic high-quality datasets derived from a variety of experimental conditions. Six of the eight studies examine the human gene expression levels during HIV infection and replication^22^,^23^,^24^,^25^,^26^,^27^ and two focus on HIV latency. The representative experiments were conducted using different cell lines or tissues, including CD4^+^ T-cells^22^,^23^,^26^,^27^ and lymphatic tissue^25^, via different experimental techniques, such as transcriptomic profiling/analysis^24^,^26^,^27^, deep RNA-seq^23^, and genome-wide mRNA expression^22^. To portrait the gene expression level during HIV latency, we selected two large-scale experimental studies^20^,^21^ to incorporate the gene expression data. Mohammadi *et al*. conducted a number of experiments to reveal the pairwise differential expression of transcripts during HIV latency and the subsequent viral reactivation following treatment of CD4+ T-cell models using different combinations of six agents, including DMSO, SAHA, CD3, IL7, DISU, and AZA^21^. The second selected dataset focused on the transcript regulation during HIV latency (latently infected CD4+ T-cells *vs.* uninfected cells) using primary CD4+ T-cell based models^20^. Three proteomic studies indicating the differentially expressed genes mediated by HIV during infection using different cell lines (CD4+ and CD8+) were also included^30^,^31^,^32^.

We combined all differently expressed genes identified in these experimental studies and further mapped them to the UniProt database^33^ to retrieve their protein products for detailed protein functional and structural annotations. Accordingly, a total of 14,318 human genes and their protein products were obtained and documented in our database. To ensure the quality of the curated entries, those genes that could not be mapped to known protein products were excluded from the database. However, in order to provide more comprehensive information, those genes that could not be identified are listed on the ‘Help’ webpage for users reference. Besides the gene expression profiles mediated by HIV, we also investigated whether the deposited entries in HIVed are HIV replication factors. Such host replication factor proteins play a crucial role in assisting HIV infection via their important biological functions in the host^12^,^15^,^34^,^35^. Therefore, to facilitate users to identify if a current protein entry has been previously described as HIV replication factor, we mapped the gene data to two mainstream experimental studies^12^,^15^ and generated corresponding identification information for each entry in the ‘Protein Information’ section. Additional information such as if the gene or protein is interferon stimulated gene, anti-viral restriction factor and/or positively or negatively associated with HIV-1 replication has been mapped to a variety of experimental studies^36^,^37^,^38^,^39^. Such information is also provided in the ‘Protein Information’ section.

**Figure 1:**
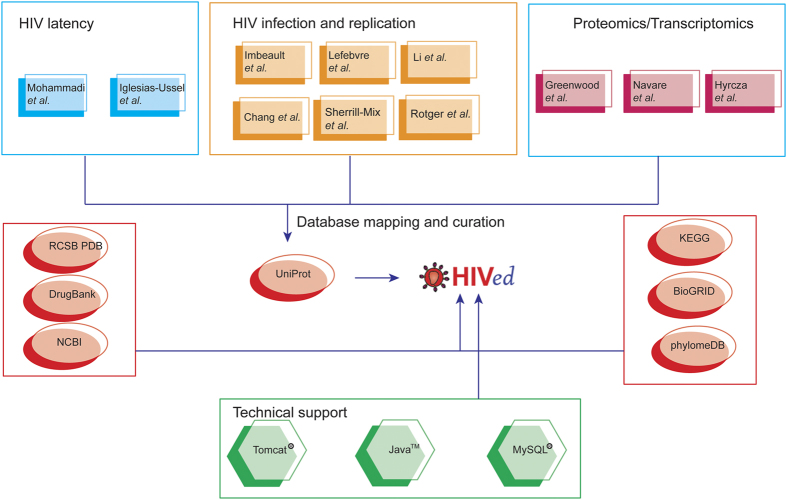
The framework for constructing HIVed including curated gene expression and proteomics datasets, cross-referenced databases and technical support.

To bridge the gap of our understanding between differentially expressed human genes during HIV infection/replication/latency and their structural and functional annotations, we further enriched the dataset by searching several other public databases and retrieving additional annotations. These include the Protein Data Bank (PDB)^40^, DrugBank^41^, HIV-1 Human Interaction database^42^, and KEGG database^43^. In addition, we also provided the accession numbers and links to BioGRID^44^ and PhylomeDB^45^ for easy retrieval of the protein-protein interaction, evolutionary information and multiple sequence alignment for each entry in HIVed, respectively. Taken together, this retrieval procedure enabled the database to integrate a variety of comprehensive biological annotations for all the entries, including protein secondary structure, drug-protein interaction, experimentally validated interaction with HIV-1 proteins, and metabolic/signaling pathway. The detailed framework for HIVed construction including datasets curation, third-party databases cross-referencing and technical support is described in Fig. 1.

### HIVed construction

HIVed was constructed using the JavaServer Pages (JSP) technique maintained by the Apache Tomcat^®^ web management system (http://tomcat.apache.org/; version: 8.0.32). We employed JavaBeans to facilitate the information transmission between the front-end webpages and the backstage of the database management system. The relational database was generated using the MySQL™ workbench (https://www.mysql.com/; version: 5.7.15) and managed by phpMyAdmin^®^ system (https://www.phpmyadmin.net/; version: 4:4.5.4.1). The host web server resides on a Linux operating system (Ubuntu; version: 16.04.1) machine with quad cores and 50 GB storage, allocated and maintained by Australia National eResearch Collaboration Tools and Resources project (Nectar; https://nectar.org.au/) and Monash eResearch Centre (https://platforms.monash.edu/eresearch/). The interactive user-interface was implemented with the help of JavaScript and other third-party JavaScript plug-ins.

### HIVed utility

**Figure 2:**
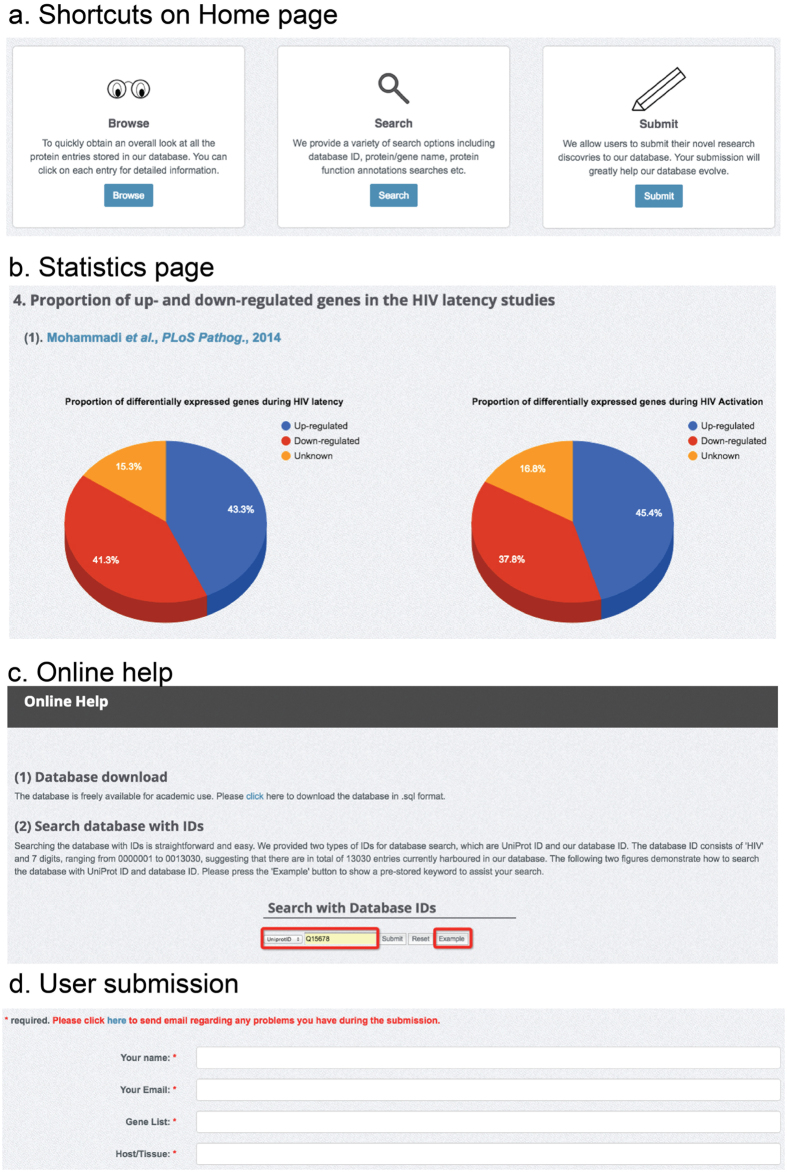
An overview of convenient functionalities provided by HIVed. (**a**) Short paths provided on the home page. (**b**) A snapshot of the database statistics page. (**c**) Online documentation for database guidance. (**d**) User submission page.

**Figure 3:**
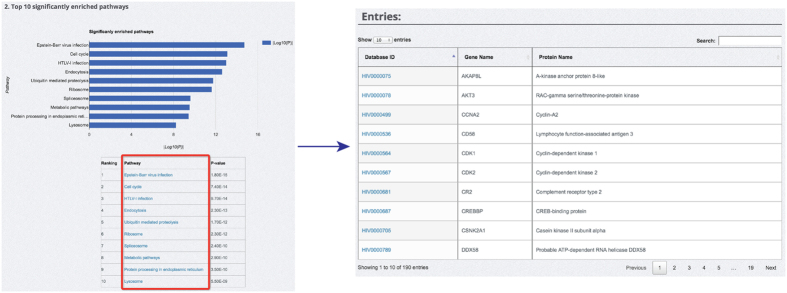
Direct retrieval of database entries involved in the significantly enriched pathways using the pathway term ‘Epstein-Barr virus infection’ as an example.

A variety of functionalities, including database search, browse and user submission, have been provided to assist the readers to use HIVed efficiently (Fig. 2). To facilitate the fast navigation of HIVed database, short paths to database browsing, search and new submissions have been provided on the ‘Home’ page (Fig. 2a). A statistics webpage is available to summarize the biological annotations documented in HIVed (Fig. 2b). Top 10 significantly enriched pathways, concluded from a statistical analysis via DAVID platform^46^,^47^ were listed on the webpage, where users can click a particular pathway to directly retrieve the entries involved (Fig. 3). For the first-time users of HIVed, we provide a step-by-step document on the ‘Help’ webpage to guide readers to explore HIVed efficiently (Fig. 2c). This page covers the detailed usage of all the functionalities including search, browsing, annotation display and user submission provided in HIVed. In addition, this page offers the link to download the whole HIVed database in the SQL format.

**Figure 4:**
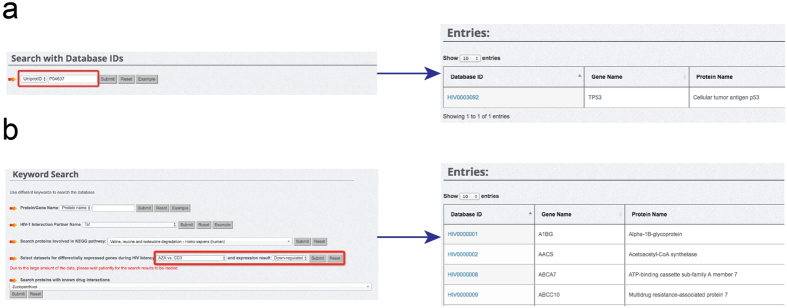
Search functions provided in HIVed. (**a**) Database ID search using UniProt ID. (**b**) Entry search using gene expression keywords.

We endeavoured to make the entry search in HIVed database convenient and straightforward. We provided two options for database search, including database ID search (Fig. 4a) and keyword search (Fig. 4b). Apart from UniProt ID, we generated independent IDs for indexing entries in HIVed. An example is available for database ID search by simply clicking the ‘Example’ button. Keyword search options, on the other hand, are flexible in order to meet different user requirements (Fig. 4b). HIVed allows users to search using a number of keywords in terms of the basic gene/protein information and functional annotations. These keywords include protein/gene name, HIV-1 interaction partner name, metabolic/signalling pathway and known protein-drug interaction annotations. Additionally, we provided a search function that allows users to promptly retrieve up- or down-regulated genes across all the experimental datasets integrated. The examples can assist users to search with pre-stored keywords. The search results will be displayed on a separate webpage with links to each detailed entry, as well as gene/protein names (Fig. 4), respectively.

**Figure 5:**
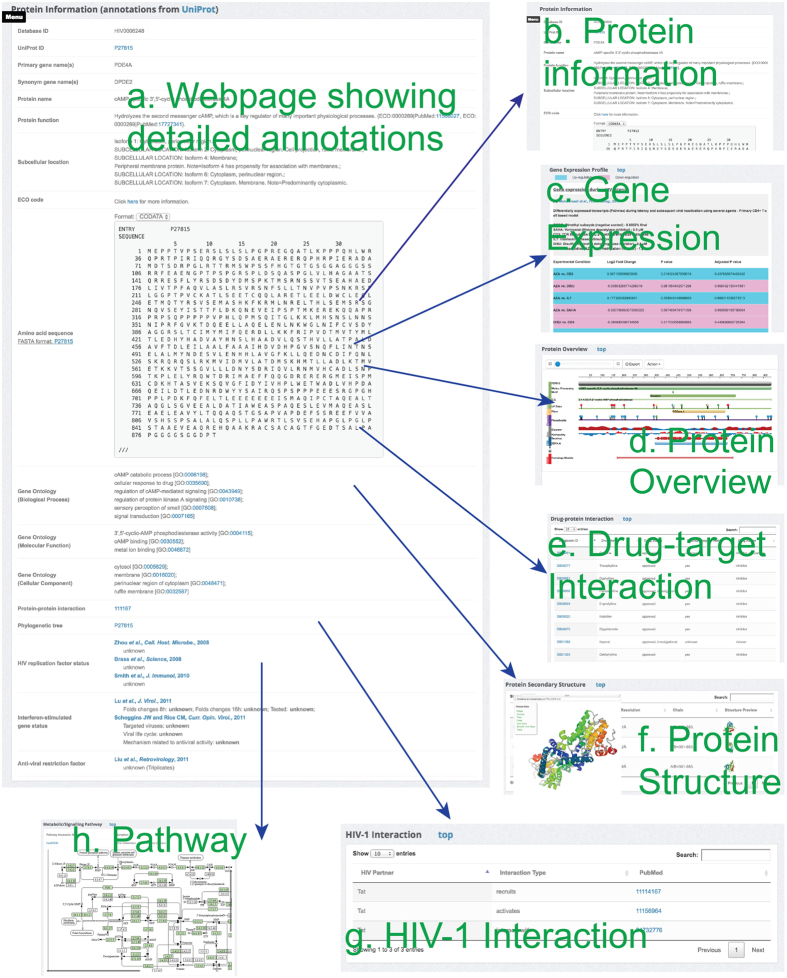
A series of screenshots showing the detailed structural and functional annotations of a particular entry in the HIVed database. (**a**) Overview of the webpage. (**b**) Protein basic information panel. (**c**) Gene expression panel. (**d**) Overview of protein functional site/domain using the Protein Feature View plug-in. (**e**) Drug-protein interaction panel. (**f**) Protein secondary structure panel with detailed portrait via the PV plug-in. (**g**) HIV-1 protein interaction panel. (**h**) Signalling/metabolic pathway panel.

After clicking the link for a particular entry, the extracted protein information, gene expression data and functional annotations will be shown on different panels of a separate webpage (Fig. 5). For each entry, seven panels including protein information, gene expression profile, protein overview, drug-protein interaction, protein secondary structure, HIV-1 interaction, and metabolic/signalling pathway, will be displayed to provide a comprehensive overview. To compare the up- and down-regulated genes across different datasets, the rows have been highlighted with different colours in the ‘Gene Expression Profile’ panel. Two widely used JavaScript plug-ins, Protein Feature View (https://github.com/andreasprlic/proteinfeatureview) and PV (https://github.com/biasmv/pv) were used to enhance the data visualization and promote users’ experiences (Fig. 5d and f). Protein Feature View plug-in offers an integrated view of proteins with their functional sites, domains and secondary structures. The PV plug-in was used to provide the protein secondary structures in a specific but fast way to the users to obtain a general understanding of protein conformational information. Detailed user instructions are available in the online manual that can guide the users to be acquainted with the use of the database.

User submission is another useful feature of the database (Fig. 2d). We would like to encourage immunologists and biochemists to submit their new discoveries related to human gene expression mediated by HIV infection, replication and latency to enhance the coverage and quality of HIVed. Additionally, entries in the database will be updated on a regular basis, by collecting entries that result from up-to-date studies pertinent to HIV latency, replication, and infection. Users can submit a new entry using the Submission module or send us an Email. A link has been provided on the submission page to assist the users to send Emails to database administrator for help. This will enable the database to keep pace with the rapid proliferation of HIV infection, replication and latency studies.

### HIVed statistics

In total, 14,318 human genes have been successfully mapped to the UniProt database with detailed functional annotations. To ensure the quality of the annotations stored in the HIVed database, those misregulated genes that could not be mapped to their protein products were disregarded. The statistics for the structural and functional annotations can be found in Table 1. One should bear in mind that the numbers listed in Table 1 should not be interpreted as straightforward biological evidence, due to the potential incompleteness of the current curated dataset in the HIVed database, and should vary upon the subsequential updates of the HIVed. In addition, we were not able to conduct statistical analysis for up- and down-regulated genes, respectively, due to the fact that the raw datasets collected in this study were derived from different tissues and experimental protocols.

### Comparison with other existing gene expression databases for HIV infection, replication and latency

As aforementioned, current databases mainly focus on the gene expression data derived from different experimental settings, such as Litchi^21^, Peachi^28^ and GuavaH^29^. These online resources provide useful information regarding the dysregulated human genes and graphical annotations showing the gene expression levels at a single time point and/or during a time course. Among these databases, GuavaH provides a variety of useful gene annotations in the perspective of HIV acquisition, viral load, durable control and HIV gene variation. It is very important to note that none of these databases focuses on the products of the dysregulated genes and their functions. Differentiating from these databases, HIVed is featured as a protein-centric knowledgebase with a special focus on the protein product of the dysregulated human genes during HIV infection, replication and latency, by combining and comparing the mainstream gene expression datasets across different experimental protocols. HIVed will enable the virologists and immunologists to easily map the human genes disturbed (or perturbed) by HIV to their functional annotations and generate hypotheses for HIV cure research.

## Conclusion

In this study, we propose an open-access HIV expression database, HIVed, to provide proteome-wide annotations of differentially expressed genes and their protein products during HIV infection, replication and latency, by systematically integrating a number of experimental studies across different protocols. By integrating a variety of biological annotations including protein basic description, Gene Ontology terms, protein confirmation, HIV-1 interaction and pathway annotations, HIVed can be seen as a comprehensive protein-centric database that allows users to input and look up genes/proteins of interest. While we acknowledge the disparate sources from which the data has been collected, comparison of such data has been difficult in the past and HIVed aims to display unbiased data reported by authors of individual studies. We anticipate this database will greatly benefit and facilitate the functional annotation and generation of novel hypotheses related to HIV-perturbed host genes and proteins with an ability to display common threads across various HIV latency and infection conditions and measurements.

## Additional Information

**How to cite this article:** Li, C. *et al*. HIVed, a knowledgebase for differentially expressed human genes and proteins during HIV infection, replication and latency. *Sci. Rep.* **7**, 45509; doi: 10.1038/srep45509 (2017).

**Publisher's note:** Springer Nature remains neutral with regard to jurisdictional claims in published maps and institutional affiliations.

## Figures and Tables

**Table 1:** Statistical summary of the structural and functional annotations for the protein entries in the current version of HIVed database.

Database annotation	Number of annotations
Number of mapped genes/proteins	14,318
Number of proteins with secondary structures	4,991
Number of signalling/metabolic pathways	310
Number of HIV-1 interactions	13,649
Number of protein-targeting drugs	4,280

Note that these numbers listed in this table could vary due to the updates of the cross-referenced databases.
